# Knowledge and practices of physicians on blood component therapy: a cross-sectional study from two tertiary hospitals in Nigeria

**DOI:** 10.4314/ahs.v21i3.32

**Published:** 2021-09

**Authors:** Esther Obi, Claudius Diette-spiff, Hannah Omunakwe

**Affiliations:** 1 Federal Medical Centre, Yenagoa, Haematology and Blood Transfusion; 2 Rivers State University Teaching Hospital, Dept of Pathology

**Keywords:** Physicians, blood component therapy, blood transfusion, blood components, knowledge

## Abstract

**Introduction:**

Comprehension of blood component therapy (BCT) has profound impact on transfusion outcomes. Variations from the standards in practices of BCT may jeopardize patient care.

**Aim:**

To assess the understanding and implementation of BCT by physicians.

**Methods:**

The study was carried out at two tertiary health care centres. It was a descriptive cross-sectional study using a self-administered, questionnaire comprising of 30 questions.

**Result:**

A total of 265 physicians responded from various clinical specialties. Physicians studied showed remarkable knowledge (98%) of BCT. Nevertheless, 92.8% of the respondents' were inclined to prescribing whole blood and the commonest reason given was ready availability at the blood bank. More than half of the respondents' have prescribed BCT with sedimented red cells and platelet concentrates being the most frequently prescribed blood components. Non-availability of blood components and cost implications were some of the identified limitations to the use of BCT.

**Conclusion:**

Majority of the physicians have a good knowledge concerning BCT. Nonetheless, there was a knowledge-practice mismatch attributable to the unavailability of the various blood components limiting optimal practice of BCT. Strategies should be formulated to overcome these identified challenges to ensure quality transfusion services in our healthcare facilities.

## Background

Blood transfusion is a very important and life-saving treatment, and a crucial component of contemporary health care. Physicians' knowledge about blood components and their demands, doses and administration, may have profound impact on patient care and outcome.

Blood component therapy (BCT) is the therapeutic use of blood components rather than whole blood to treat a specific deficiency, avoid volume overload and minimize reactions to blood products that are not needed1. Blood components includes red cell concentrates (RCCs), fresh frozen plasma (FFP), platelet concentrates (PCs) and cryoprecipitate^1^.

Commonly, RCCs and other blood components are obtained by centrifugation methods with programmable centrifuges and blood components separators (automatic or not) after whole blood collection, or through apheresis process^2^. In Sub-Saharan African, donor collection by apheresis and component separation using centrifugation methods are often out of reach. Transfusion services in those jurisdictions have neither the financial resources nor the technical capacities to implement these processes. Limited financial resources, shortage of trained staff and the poor electrification of these settings are the main constraints. Red cell concentrates are prepared by simple gravity, sedimentation in most of these countries including Nigeria^3^.

Most transfusion decisions are made by physicians with exposure in transfusion medicine from only undergraduate medical courses only and this knowledge has been observed in studies to be insufficient^4^. Majority of blood banking training barring transfusion medicine post graduate programme, is given in pathology residency programs. Unfortunately, these specialists are not always the bedside providers of blood transfusion^5^. Physicians are required to have a basic knowledge of transfusion medicine to prescribe the right components for the right indications and practise its appropriate use^6^.

In most developing countries like Nigeria, the demand for blood far outweighs the supply^7^,^8^. There is thus, a need to optimise the use of blood component therapy by highlighting that one donation can be used for three transfusions. Therefore, adequate knowledge of blood component therapy by physicians may aid with the problems related to blood shortage and improve the quality management of patients who require blood transfusion.

The aim of this study was to assess the understanding and implementation of blood component therapy amongst physicians practising at two tertiary hospitals in Nigeria. There are limited studies assessing the knowledge and practice of blood component therapy among physicians in Nigeria. The findings of this study may be used to address the shortcomings in blood component therapy through continuing medical education and training to improve the quality of care of patients.

## Methods

This was a descriptive cross-sectional survey using a self-administered questionnaire. A total of 265 physicians participated in this study.

This study was conducted in two tertiary care centers (University of Port Harcourt Teaching Hospital, Rivers State and Federal Medical Centre Yenagoa, Bayelsa State) within the South-South geopolitical zones of Nigeria. Nigeria is divided into six geopolitical zones (North-Central, North-East, North-West, South East, South-South and South-West) inclusive of states with similar ethnic groups and political history. States in the South-South zone are: Akwa-Ibom, Bayelsa, Rivers, Delta and Edo. The blood banks in these healthcare institutions provide blood services to all specialty and super-speciality wards.

The study was approved by the Federal Medical Centre, Yenagoa ethics committee and informed consent forms were signed by all participants.

Inclusion criteria were physicians from those specialities, where blood transfusion events are common like obstetrics and gynaecology, surgery, medicine, anaesthesia and paediatrics. Exclusion criteria includes: house Officers who have not completed at least two clinical rotations of approximately 24 weeks; physicians who were not actively working in clinical departments.; physicians from pathology who receive transfusion medicine training as their curriculum and who would have responded significantly better than others.

The questions were set by experienced transfusion medicine specialists at the Haematology department and reviewed by specialists of other departments for its content and clarity. A pilot test of the questionnaire was undertaken in hospital using a random sample of physicians from another tertiary hospital not under study who responded that the items were clear, important and understandable.

The survey consisted of 30 questions which were designed to address physicians' knowledge and clinical use of blood components including the limitations observed in the course of their practice. The questionnaire was offered to as many physicians as possible in the above mentioned departments without disturbing the routine. Survey was conducted only once in every department. All participants from one department were assessed simultaneously. Questionnaires was collected immediately after completion. Whole survey in all the above mentioned departments was conducted in three days in no particular order. Multiple-choice questions were used in this study to avoid the problems associated with scoring open-ended responses. The maximum possible score for the individual was 30 as each correct response was awarded one point. Some of the multiple choice questions had multiple answers. Each correct response was awarded one point, for questions with multiple answers, each option was also awarded one point respectively. A participant who gets all the options in a question wth multiple answers is awarded a full one point.

The data was presented using descriptive statistics (frequency and percentages). Chi-square analysis was used to assess the differences in the distributions of the variables. All analysis was done with the SPSS v25 software (IBM, USA) at a 95% confidence interval and a p-value of 0.05 was considered significant.

## Results

A total of 265 physicians participated in this study – out of which 192 (72.1%) were residents, and 9 (3.4%) were consultants. Majority of the participants were within the age range of 31 – 40 years (table 1).

**Table 1:** Demographic Distribution of Respondents

	Age Groups	Frequency (n = 265)	Percent
**Age Groups**	20–30 years	41	15.5%
	31–40 years	161	60.8%
	51 – 60 years	55	20.6%
	41–50 years	8	3.1%

**Gender**	Female	117	44.3%
	Male	148	55.7%

**Designation**	Consultant	9	3.4%
	Medical officer	7	2.6%
			21.1%
	Resident	192	72.5%
	Other	1	0.4%

**Years of** **Practice**	<1 year	39	14.4%
	1–5 years	90	34.0%
	5–10 years	68	25.8%
	Above 10 years	68	25.8%

Internal medicine physicians were the highest proportion of respondents from the 168 participants who indicated their field of specialty as seen in figure 1.

**Figure:**
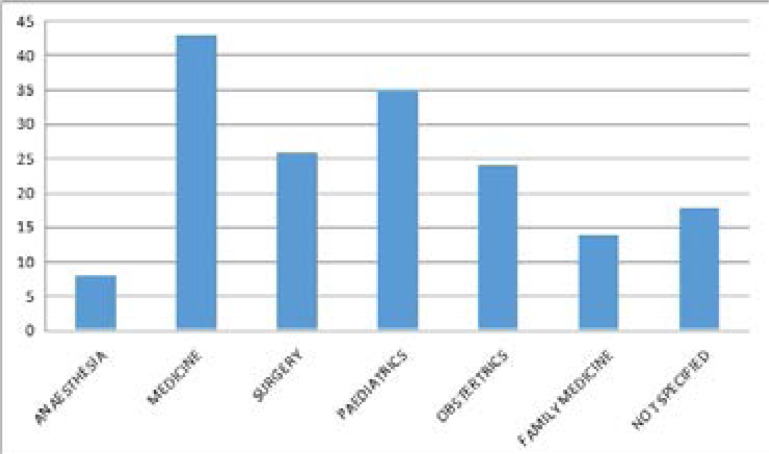


### Knowledge of Blood Components

Ninety-eight percent of respondents indicated they had heard about BCT. The two major sources of information were from undergraduate lectures (157) and personal reading (127) as shown in figure 2. None of the respondents correctly identified all the components. Table 2 shows the most correctly identified blood components were fresh frozen plasma (94%), platelet concentrate (90.9%) and sedimented red cells (86.8%). Table 2 also identifies fresh whole blood (75.1%) as the most wrongly identified answer from the respondents. A greater number of the participants indicated that voluntary donation is the best source of blood donation. As shown in table 3. The most commonly identified benefits of BCT were: prevention of volume overload 222(83.8%); specific form of therapy 221 (83.4%), and better patient management 207 (78.1%). Only 97(36.6%) of respondents identified BCT as cost effective where few donors are available as shown in table 4. A high proportion of the respondents (92.8%) would rather prescribe whole blood. One of the highlighted reasons was the ready availability of whole blood at the blood bank.

**Figure:**
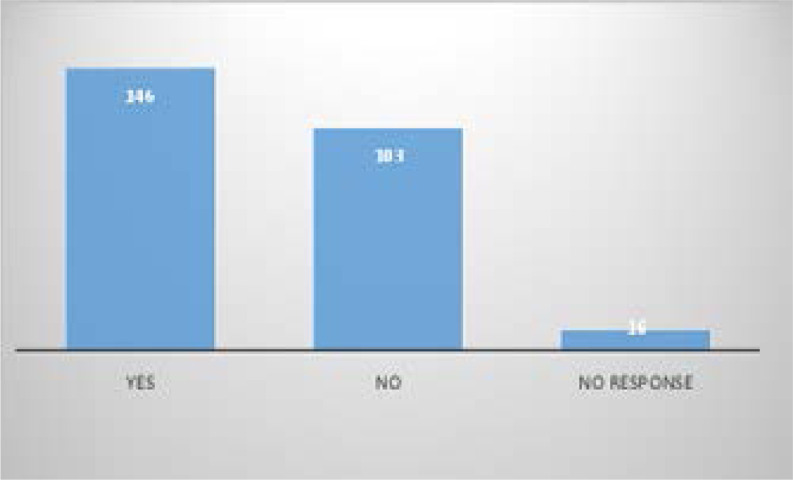


**Table 2:** Blood Components that can be transfused

Components	Frequency	Percentage*
Sedimented red cells	230	86.8%
Fresh whole blood	199	75.1%
Platelet concentrate	241	90.9%
Fresh frozen plasma	249	94.0%
Cryoprecipitate	206	77.7%
Plasma expanders	65	24.5%
All of the Above	15	5.7%

**Table 3:** Best source of donor

Best Source of Donor	Frequency	Percent
Voluntary	197	74.2%
Self-donor	49	18.6%
Remunerated donor	3	1.0%
Replacement donor	6	2.1%
Other	10	4.1%
**Total**	**265**	**100.0%**

**Table 4:** Benefits of BCT

Benefits of Blood Component Therapy	Frequency	Percent*
Prevents volume overload	222	83.8%
It is a more modern form of therapy	165	62.3%
Prevents the formation of alloantibodies	110	41.5%
It is a more specific form of therapy	221	83.4%
Gives room for better management of our patients	207	78.1%
To correct hypovolemia.	52	19.6%
Cost effectiveness for where few donors are available.	97	36.6%

### Practice of BCT

More than half of the respondents indicated that they had prescribed BCT for the treatment of their patients. Sedimented cells 129(48.7%), platelet concentrate 105(39.6%) and fresh frozen plasma 97(36.6%) were the most frequently prescribed blood components. The major indications were severe anaemia 201(75.8%) and thrombocytopenia 153(57.7%) respectively. Some of the limitations to component therapy mentioned to the practice of BCT included; non-availability of components at the blood bank (78.5%) and cost implications (62.6%).

Majority of the physicians admitted difficulty in getting donors and some of the reasons highlighted were blood incompatibility for available donors and unwilligness of family members to donate as shown in table 5.

**Table 5:** Limitations of the use of BCT

Limitations to the use of BCT	Frequency	Percent
Cost implication	166	62.6%
Awareness not enough	155	58.5%
Non availability in the blood bank	208	78.5%
Non availability of component preparation materials	131	49.4%
Component machine breakdown	78	29.4%

## Discussion

One of the key points determining the efficiency of any blood transfusion service is the knowledge level of the physicians using the service.

This study showed that major information on blood component therapy was from undergraduate training. With the changing growing global trends in transfusion medicine, basic knowledge from undergraduate studies may not be sufficient to ensure standard medical practice.

Most of the physicians were able to correctly identify the different types of blood components. However, fresh whole blood was wrongly identified as a blood component by 199 participants. Fresh whole blood has been variously defined as blood collected at less than 4 hours (ultra-fresh), 24 hours, 72 hours and stored at room temperature^9^. Its ushas been advocated in cardiac surgery, burns and, massive transfusion, particularly in the military setting^10^,^11^. Potential risks have been reported with the use of fresh whole blood therefore, its use is best limited to clinical trials and situations where there is life-threatening bleeding, and blood component therapy is unavailable^12^. Physicians should know the different types of blood components and their doses to be able to make appropriate prescriptions.

BCT has been reported to be beneficial in protecting the national inventory at times of national blood shortage^13^. This is because several patients can potentially benefit from components derived from one unit of donated blood. This is contrary to the findings in this study where less of the physicians believe that BCT is cost effective where few donors are available.

Majority of the physicians prefer to prescribe whole blood despite showing a good knowledge of the benefits of BCT. This is in congruence with of findings in the literature which showed most transfusion recipients receive whole blood^14^,^15^. The physicians' preference to whole blood is probably because of ‘availability’ reasons. Non-availability of the components was identified as one of the limitations to the practice of BCT as reported in most studies^16^,^17^. Sedimented cells was the most prescribed blood component as seen in other studies^17^.

The study also shows that most physicians agree that voluntary blood donation still remains the best source of blood donation. However, there is paucity of voluntary donors in most developing countries including Nigeria as seen in our study where more than half of the doctors identified the absence of voluntary donation as one of the difficulties encountered in blood donation^18^,^19^.

Replacement/family donors is reported to be the the most prevalent source of blood donation^20^. This finding has been linked to the fact that it may actually cost less to procure and is also well adapted to the extended family support system of many Nigerian and African communities^20^. Some of the limitations to this practice as also observed in this study includes blood group incompatibility or refusal to donate by the family donors.

## Limitations of study

The generalizability of this study may be limited since study participants consisted of physicians from two tertiary hospitals.

## Conclusion

Our study has shown that majority of the physicians in Nigeria have a good knowledge regarding blood component therapy. However, availability of the different components limits optimal practice of blood component therapy.

## Figures and Tables

**Table 6:** Difficulties encountered in getting blood donors

Difficulties in getting donors	Frequency	Percent*
Donor available was blood-group incompatible	158	59.6%
Donor available had transfusion transmissible disease	107	40.4%
Inadequate PCV of donor	119	44.9%
Inadequate platelet count of donor	31	11.7%
No family member around to donate	151	57.0%
Family member available unwilling to screen	133	50.2%
Cost of paying a donor	116	43.8%
No voluntary donor	144	54.3%
